# Integrated clinical case discussions – a fully student-organized peer-teaching program on internal medicine

**DOI:** 10.1186/s12909-022-03889-4

**Published:** 2022-12-01

**Authors:** Johannes Reifenrath, Nick Seiferth, Theresa Wilhelm, Christopher Holzmann-Littig, Veit Phillip, Marjo Wijnen-Meijer

**Affiliations:** 1 School of Medicine, Technical University of Munich, TUM Medical Education Center, Ismaninger Str. 22, 81675 Muenchen, Munich, Germany; 2 Department of Nephrology, Hospital Klinikum Rechts Der Isar of the Technical University of Munich, Munich, Germany; 3 Department of Internal Medicine II, Hospital Klinikum Rechts Der Isar of the Technical University of Munich, Munich, Germany

**Keywords:** Undergraduate Medical Education, Clinical Skills, Instructional Design, Peer-to-Peer, Problem-based/Clinical Case Discussion

## Abstract

**Background:**

In response to students´ poor ratings of emergency remote lectures in internal medicine, a team of undergraduate medical students initiated a series of voluntary peer-moderated clinical case discussions. This study aims to describe the student-led effort to develop peer-moderated clinical case discussions focused on training cognitive clinical skill for first and second-year clinical students.

**Methods:**

Following the Kern Cycle a didactic concept is conceived by matching cognitive learning theory to the competence levels of the German Medical Training Framework. A 50-item survey is developed based on previous evaluation tools and administered after each tutorial. Educational environment, cognitive congruence, and learning outcomes are assessed using pre-post-self-reports in a single-institution study.

**Results:**

Over the course of two semesters 19 tutors conducted 48 tutorials. There were 794 attendances in total (273 in the first semester and 521 in the second). The response rate was 32%. The didactic concept proved successful in attaining all learning objectives. Students rated the educational environment, cognitive congruence, and tutorials overall as “very good” and significantly better than the corresponding lecture. Students reported a 70%-increase in positive feelings about being tutored by peers after the session.

**Conclusion:**

Peer-assisted learning can improve students´ subjective satisfaction levels and successfully foster clinical reasoning skills. This highlights successful student contributions to the development of curricula.

## Background

The SARS-CoV2-pandemic´s strain on medical schools has been hard [1–3] since many stakeholders in medical education are both caregivers and instructors. With limited staff available for teaching [4] and reduced on-campus presence, many classes were moved to emergency remote teaching courses [5, 6]. Emergency remote teaching is the “alternate delivery mode due to crisis circumstances” as opposed to well-planned online teaching [7].

At Technical University of Munich (TUM) most lectures, seminars, and bedside teachings werecanceled or moved to emergency remote teaching in the spring semester of 2020. Within the student council, the notion quickly gained traction that a peer-assisted learning (PAL) program ought to be established to alleviate pressure on faculty staff while providing students with a safe environment for the acquisition and training of their clinical reasoning skills.

Several universities have promoted PAL programs. It refers to the “development of knowledge and skill through explicit active helping and supporting among status equals” [8]*.* Benefits of PAL are i) a similar knowledge base and an understanding of obstacles while studying (cognitive congruence) [9, 10], ii) a positive learning environment void of complicated student-instructor relationships due to similar social status (social congruence) [9, 10] and iii) relieving pressure on faculty staff [11]. PAL has been employed in teaching anatomy, physiology, and biochemistry [12], as well as communication [13], physical examination [14], and other procedural skills [15, 16]. There students have been shown to assume the roles of lecturers [17], clinical or practical teachers [18], mentors [19], learning facilitators [20, 21], role models [21], and assessors [22]. In our study, we wish to introduce a curriculum that was fully designed, delivered, and evaluated by undergraduate students based on the Kern Cycle [23] with minimum intervention by faculty staff. We thus empower students to holistically assume all of the twelve roles of a teacher as proposed by Harden and Crosby in 2000 [24].

Targeted at students in the clinical phase of their studies, we developed the novel *Integrated Clinical Case Discussions* (ICCD) that emphasize the training of clinical reasoning skills that are at the heart of the recently released second edition of the competence-based German Medical Training Framework (GMTF) [25]. In accordance with the GMTF three central learning objectives were identified: i) transfer of clinical knowledge, ii) fostering of diagnostic management skills, and iii) enabling students to discuss findings and procedures in a team. Clinical Case Discussions (CCD) have been shown to enhance clinical and scientific reasoning skills [20, 26], self-directed learning [26], and exchange with colleagues [27].

This study seeks to explore whether a peer-moderated clinical case discussion can improve students´ subjective satisfaction level with learning opportunities in case of emergency remote teaching.

## Setting and participants

For their studies of internal medicine students at TUM attend two series of lectures in two consecutive semesters: In the spring semester of their first clinical year, there is a series of lectures on the cardiovascular and hematologic systems. In the subsequent fall semester, they hear a series of lectures on nephrology, gastroenterology, and endocrinology. Students are routinely requested to evaluate all lectures on a five-point Likert scale. When in the spring semester of 2020 all lectures were moved to an emergency remote teaching format, the mean evaluation of lectures on internal medicine dropped by 1.44 points as opposed to the six years prior (from 1.96 to 3.4, where 1 denoted the greatest and 6 the lowest level of satisfaction).

To provide their peers with an additional opportunity to review the lectures´ content, three students initiated the peer-moderated Integrated Clinical Case Discussions. In the ICCDs we applied a lecture´s content to a patient´s case with special emphasis on diagnostic and management skills in accordance with GMTF level 2 (i.e. clinical reasoning skills).

**Fig. 1:**
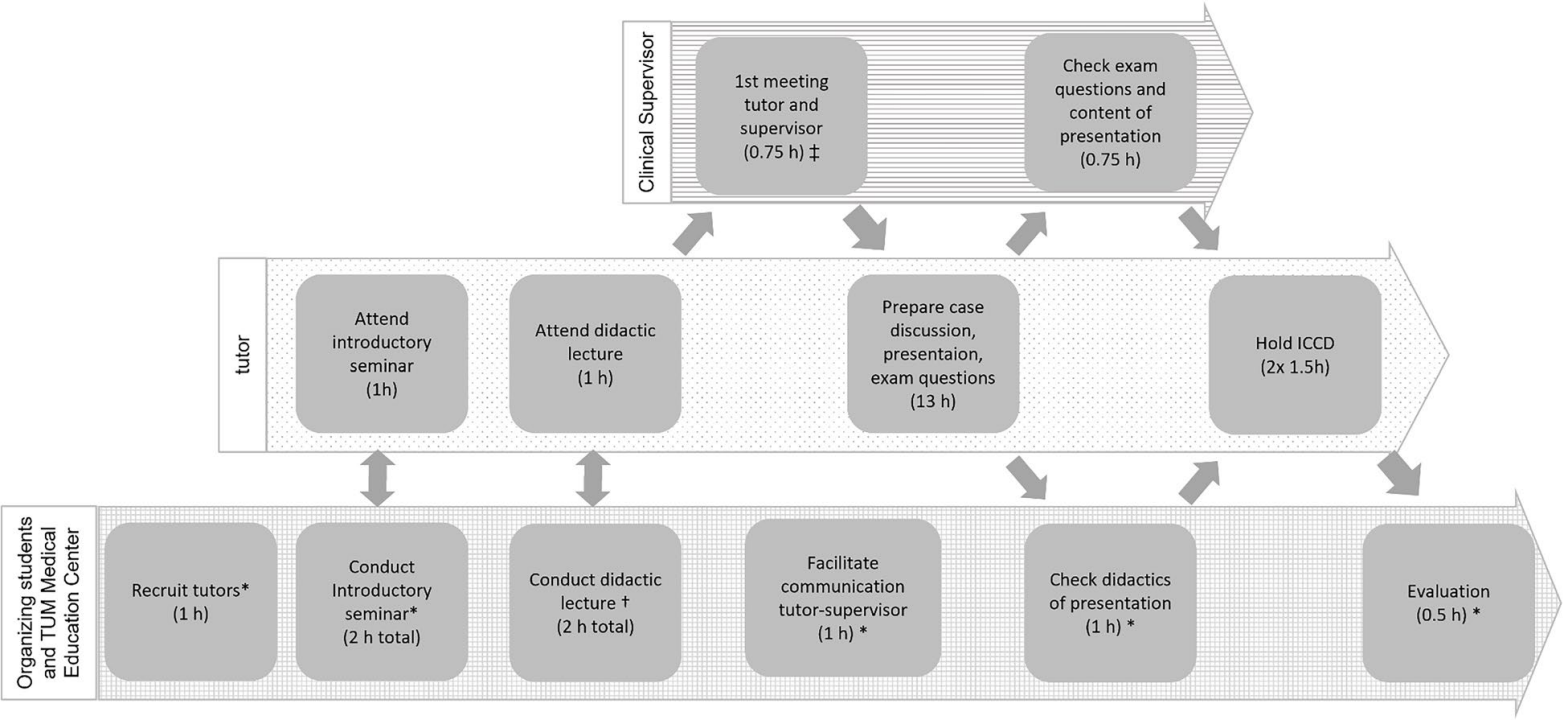
Workflow for the preparation of one ICCD session. Three parties are involved in the preparation and implementation of an ICCD session: an administrative unit consisting of the organizing undergraduate students (*) and the TUM Medical Education Center (†) (bottom row), tutors (middle row) and clinical supervisors (top row). Their respective tasks are indicated at the relative time points for the preparation of one ICCD. The allotted time frame for each task per one ICCD session is included in round brackets. For their first meeting tutors and supervisors are provided with a checklist (‡), i.e. to i) define content-focal points, ii) select an appropriate clinical case iii) define a clinical skill essential for the successful completion of the case, and to iv) provide the tutor with important clinical findings (e.g. laboratory findings, images)

We prepared ICCDs for 12 topics in the fall semester of 2020 and 12 topics in the spring semester of 2021. For each topic we allocated two 90-min sessions in the week immediately following the general lecture on the topic. We were able to offer one face-to-face and one online tutorial for 11 topics. Due to hygiene regulations, the remaining 13 topics were discussed exclusively online twice a week. The time resources needed for one tutorial included i) 18 h for the tutor to prepare and hold the ICCD, ii) 3.5 h for the organizing students to recruit and mentor tutors as well as to evaluate and advertise the sessions and iii) 1.5 h of supervision by the physician (Fig. 1). The remuneration was 250€ per tutor and tutorial and 246.66€ for each organizing student per month (In the first year: authors JR and NS—15 months, TW—5 months. This was later reduced to one organizing student only.). Tutors were trained and supervised by specialist physicians as part of their regular teaching duties (1.5 h per session). Physicians were not reimbursed by the ICCD team. ICCDs were completely voluntary. We advertised ICCDs through weekly email alerts and a note in students´ schedules.

## Methods

### Conception of the didactic concept

**Fig. 2:**
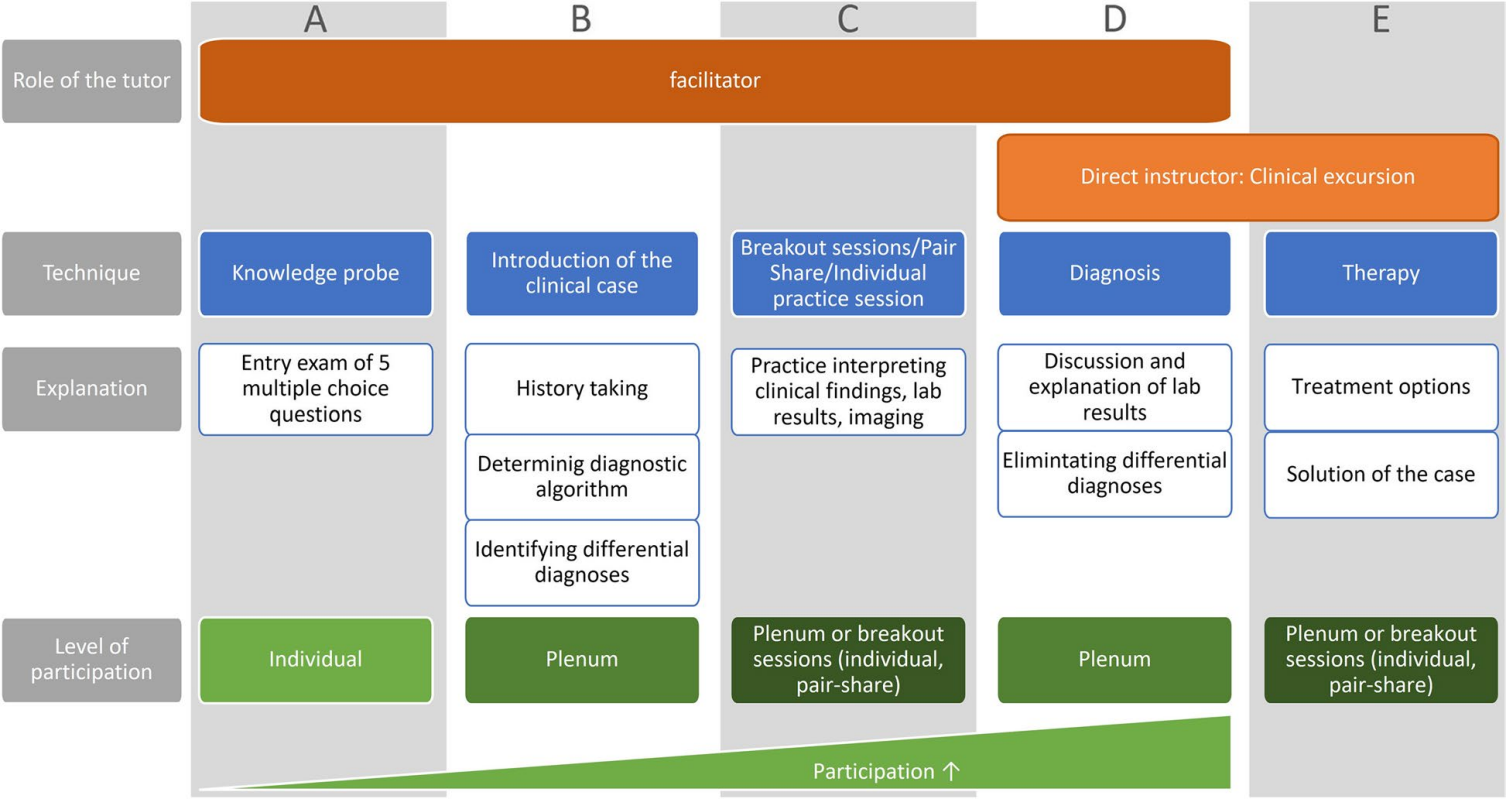
Typical outline of an ICCD session. We modified Linsenmeyer´s approach to stimulating interaction between students (Linsenmeyer, 2021). One ICCD session propagates along the x-axis from left to right. Several layers along the y-axis indicate the roles a tutor assumes at each time point, the teaching techniques they employ (examples provided below), and the level of interaction this is likely to be incentivize between tutees. A: Each session starts with a knowledge probe intended to activate students´ prior knowledge by asking five multiple-choice questions that participants must solve individually and anonymously. As indicated by the green triangle at the bottom of the figure this requires only a minimum level of interaction between students. B: Subsequently, tutors introduce the session´s clinical case and moderate a plenum discussion in which participants collectively take a patient´s history, determine an appropriate diagnostic algorithm, and list differential diagnoses. This gradually raises the level of interaction (upward slope of the triangle). C: In the next stage participants are assigned to break-out groups of two to four students in which they practice interpreting patient-specific clinical findings, lab results or different image modalities. Tutors switch from group to group to help if needed. D: Finally, the breakout groups meet back in the plenum and discuss their findings and differential diagnoses under the tutor´s moderation. We rated this as the most demanding level of interaction as it requires students to present in front of a larger group. At this point, tutors are oscillating between facilitating the discussion as different groups present their findings and providing direct instruction when explaining the meaning behind lab results/images, etc. Under the tutor’s guidance differential diagnoses are eliminated and the final diagnosis emerges. E: Lastly the tutor outlines treatment options. Due to time constraints, this was predominantly done in direct instruction

ICCDs followed cognitive learning theory. Each session was set as an interactive problem-based learning scenario (*Clinical Case Discussion*), that facilitated learners´ active participation to organize and conceptualize information [28]. To prompt students to access pre-existing knowledge ICCD sessions started with a voluntary entry-exam of five multiple-choice questions. The prefix *Integrated* reflects the close alignment of student-led tutorials and the lectures conducted by faculty staff. ICCDs did not seek to introduce new facts but to offer a platform for reviewing and applying the lecture’s contents to a clinical case. Each ICCD comprised two clinical cases in which at least one skill apart from history taking was trained (usually the interpretation of laboratory findings). Tutors and lecturers chose a clinical case from the lecturer´s clinical experience that matched the lecture. Tutors then prepared a powerpoint presentation (Microsoft Corp., Redmond, Washington, USA) to facilitate the case discussion, which was checked by the lecturer for medical content and by the organizing students for the didactic concept. Tutors delivered online sessions through a university zoom account (Zoom Video Communication Inc., 5.7.7, San Jose, California, USA) and—if under the Covid-regulations permissible—face-to-face in the lecturing hall. We instructed tutors to follow a modified version of Linsenmeyer´s approach [27] (Fig. 2). Briefly, tutees´ participation and teamwork were gradually increased by moving from anonymous multiple-choice questions to group discussions in breakout rooms and finally to discussing the ideal diagnostic procedures in the plenary session. An example of one case can be found in the supplementary material S1.

### Recruitment and training of tutors

Tutors were recruited from the student body of those students who had completed the lecture on internal medicine and passed the exam. The recruitment process was based on Engel´s approach [17] and included a publicly shared application form and a job interview in which a shared decision was made on the topic best suited to the tutor´s interests and experience. A standardized curriculum was designed for tutors and delivered by a joint group of clinicians, the TUM Medical Education Center, and the organizing students who provided the impetus for ICCDs (Fig. 1). Mandatory training consisted of an introductory seminar on the ICCD´s didactic concept and a lecture on how to teach clinical reasoning skills and stimulate group interaction. Tutors then prepared their tutorial with their clinical supervisor as described above.

### Questionnaire

Tutee evaluations were collected online at the end of each session using EvaSys V8.1 (evasys GmbH, Lueneburg, Germany). The survey comprised 50 self-report questions (supplementary material S2). Items were rated on a five-point Likert scale from 1 (strongly agree) to 5 (strongly disagree). For selected items, we also asked open-ended questions.

The underlying concept of the evaluation tool was modeled on the Student´s Evaluations of Educational Quality Questionnaire (SEEQ), a validated and reproducible evaluation tool proposed by Marsh in 1982 [29]. Designed for summative assessment of faculty-administered teaching, the SEEQ had to be adapted to our specific needs. We adopted evaluation items “I Learning/Value”, “IV Group Interaction” and all applicable items of “III Organisation” and “V Individual Raport”, yet omitted items VI-IX, since participation was completely voluntary, and examinations were not part of the ICCDs. Following the SEEQ category “I Learning/Value” we compared students´ subjective assessments of gain in knowledge, skill, motivation, and overall grade [30] after attending only the general lecture with attending both lecture and ICCD. We excluded SEEQ-section “II Enthusiasm” since tutors would have to proactively volunteer to teach in addition to their regular workload. Instead, we wanted to measure tutors´ performance as levels of cognitive congruence and educational environment. The tutor intervention profile by De Grave [31]and the Student Course Experience Questionnaire by Paul Ginns [32] reflected the aforementioned categories in more detail than the SEEQ and served as a reference. (Appendix Table 1). We also asked tutees to identify roles the tutor had assumed for them as proposed by Bulte et al. [21]. Learning outcomes were assessed as comparative self-assessment (CSA) for aggregated data [33]. The questions´ wording was based on the GESIS survey guidelines [34].

We handed tutors a short survey that asked them to rate the helpfulness of the introductory seminar, their understanding of the overall concept, and their difficulties in preparing the ICCD and enjoyment of the process on a five-point Likert scale.

### Statistical analysis

We analyzed data using SPSS Statistics for Windows version 27 (IBM Corp., Armonk, New York, USA) and Microsoft Excel (Microsoft Corp., Redmond, Washington, USA). We included all surveys that had answers to more than 50% of all questions. If a student visited multiple sessions, only their first response to each question was included in the analysis. Learning outcomes and shifts in attitude toward peer teachers were computed as the CSA-gain as proposed by Raupach et al. (2011) [33]. Briefly, at the end of each session students were asked to retrospectively rate their expertise in the item before and after attending an ICCD session. The average net increase in self-assessment was then displayed as a percentage-wise increase over the average initial self-assessment. Qualitative, descriptive data were measured on a five-point Likert scale and analyzed using mean, mode, and standard deviation. Testing for statistical significance was performed using a two-tailed exact Chi-Square Test for categorical variables. Mann–Whitney-U-test was used for the comparison of metric variables with non-normal distribution between two groups (learning outcome). A p-value of 0.05 was chosen a priori. Effect size was calculated using Cramer´s V for descriptive data and correlations were computed using Spearman Correlation. Cronbach´s alpha was computed to test for internal consistency for the categories “cognitive congruence” and “educational environment”. Answers to open-ended questions were analyzed according to qualitative content analysis by Mayring [35]. Author JR developed the major categories deductively based on probable answers and supplemented them with subcategories inferred from students´ final responses. Another author, NS, checked categories for traceability. Finally, a category tree with specific anchor examples and defined subcategories emerged. The frequency of items and total number of student comments were recorded.

## Results

### Demography

In the fall semester of 2020, a total of 335 students enrolled in the general lecture, 149 (44.5%) of whom attended at least one ICCD session. In the subsequent spring semester, 334 students enrolled in the general lecture and 237 (71.0%) took part in at least one ICCD session. Some tutees attended multiple sessions throughout the semesters. In sum, we counted 273 student attendances in the first and 521 in the second semester, respectively.

We received evaluations from 32.4% of all participants (*n* = 125). 91 (72.8%) tutees were aged 25 or under and 96 tutees (76.8%) identified as female. This approximately reflected the general student population (female/male: 65/35; mean age: 24 years). Questionnaires without informed consent were excluded from further analysis.

We employed 19 tutors for the implementation of 48 ICCD sessions covering a total of 24 topics. Eleven (57.8%) of those tutors identified as female and 12 (63.1%) had gained previous experience in front-line tertiary teaching.

### Acceptance of ICCD

**Fig. 3:**
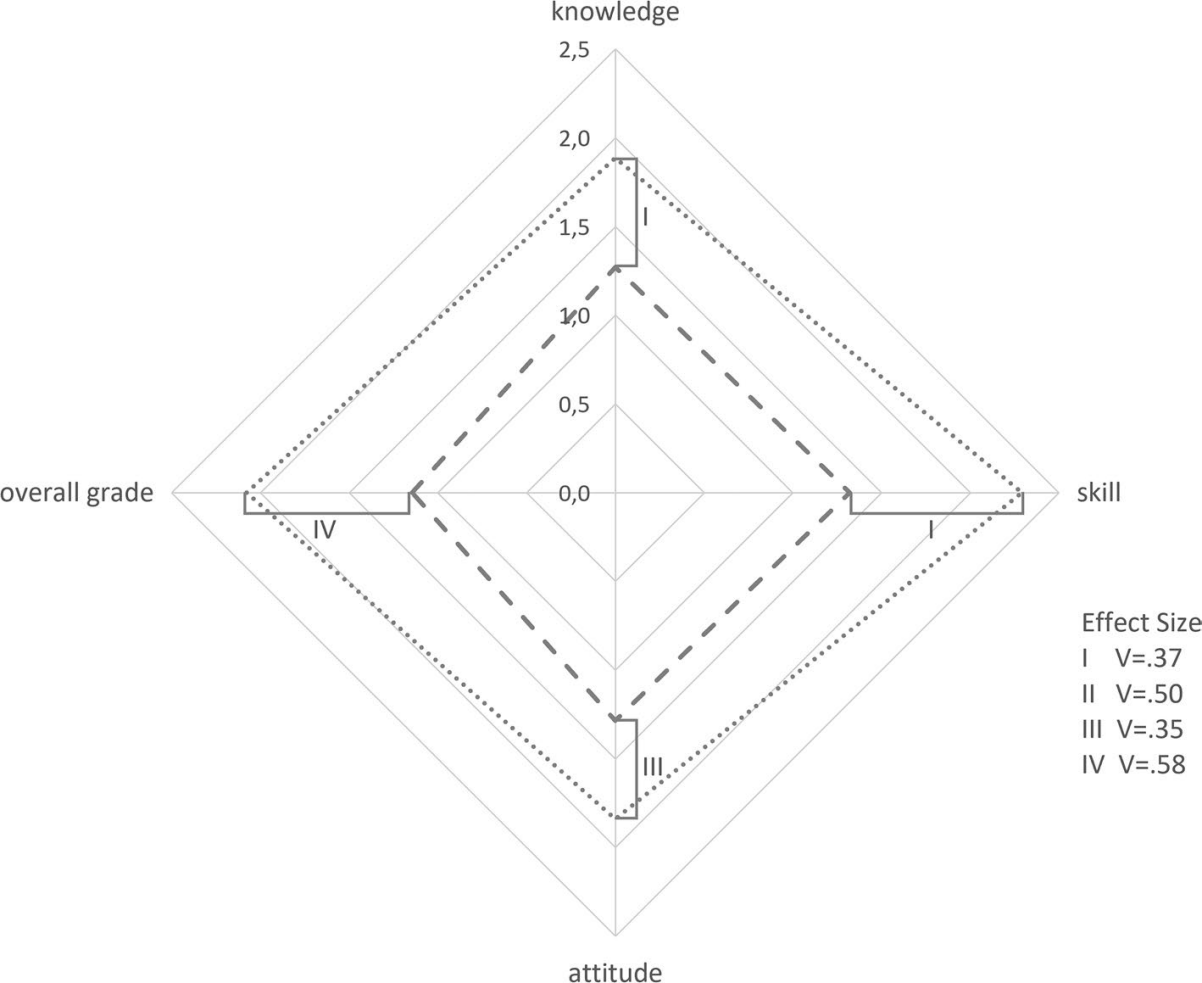
Evaluation of ICCD vs. lecture. A Kiviat diagram representing students´ mean subjective assessment after attending lectures alone (round dots) and after attending both lectures and ICCD (long dashes) in categories knowledge, skill, attitude, and overall grade each represented on one of the axes of the diagram. Students were asked to rate their gain in each of the categories for both tutorial and the respective lecture on a five-point Likert scale with 1 denoting the greatest and 5 the lowest degree of satisfaction. Questionnaires were administered immediately after each tutorial. Tutorials took place one week after the general lecture. All differences are significant (*p* < .01). Effect size was calculated using Cramer´s V

The nature of the ICCD being an add-on to the standard curriculum, we aimed to create additional value to the core curriculum that could not be attained with lectures and seminars alone. Evaluation of the ICCD shall therefore be displayed in direct comparison to the corresponding lecture (Fig. 3). ICCDs were generally rated as excellent and significantly better than lectures for all categories: knowledge, skill, attitude, and overall grade. Effect size was greatest for overall grade (V = 0.58; *p* < 0.01) and smallest for gain in knowledge (V = 0.37; *p* < 0.01) in ICCDs as opposed to the lecture. We observed that gain in knowledge correlated with gain in skills (r = 0.56; *p* < 0.01) and overall evaluation of the ICCD session (r = 0.61; *p* < 0.01).

When asked how comfortable tutees felt about being tutored by peers for an ICCD, tutees indicated a 70% increase in positive feelings after the intervention (CSA gain = 69.57%, *n* = 111).

We received 57 answers to the open-ended questions on satisfaction and improvement suggestions (Table 1). In these answers, a total of 123 text segments (k) were identified and grouped into four categories. Most test segments praised the general format of the ICCD (k = 45). The second most frequent category included individual feedback on tutors (k = 40). The third category addressed learning value (k = 24) and the last category included improvement suggestions (k = 14).

**Table 1:** Qualitative Content Analysis of Open-Ended Questions on Student Satisfaction

Category	k*	Example
**Subcategory**		
General Format	45	
general appraisal of the tutorial	27	“ICCD sessions are the best course this semester […]”
interaction rated as positive	9	“[…] no other class has that level of interaction […]”
clinical cases rated as useful for practicing lab results	5	“Discussing lab results to this extent was great!”
appropriate group size	2	“Using virtual break-out groups was a good idea.”
positive atmosphere incentivizes participation	2	“I enjoyed the tension-free atmosphere, that encouraged me to participate, even if my contributions had to be corrected”
Feedback on Tutors	40	
expression of gratitude	15	“Many thanks to the tutor!”
tutor exhibits good didactic skill	12	“[…] As a tutor for paramedics myself I am always surprised by the didactic skills that [ICCD-]tutors exhibit.”
explicit praise of the tutor (not further specified)	8	“Excellent tutor! […]”
tutor is highly motivated	5	"Thanks a lot for the effort and motivation that goes into preparing the tutorials.”
Value	24	
participants learned a lot	15	“Great seminar, with many take-home lessons.”
call for preserving ICCD in the next semesters/expanding to other subjects	9	“[…] Please continue [ICCD] at all costs!”
Improvement suggestions	14	
duration too long or too short	5	“[…] a bit long.”
the online tool (zoom) needs to be optimized for discussion	3	“[…] regrettably discussions via Zom tend to be a bit lame[…]”
tutees request factsheets and slides before the ICCD sessions	2	“A factsheet on history taking and physical examination would be appreciated […]”
more self-directed, active learning requested	2	“ […] less content, more time for active learning, please.”
more interaction requested	2	“Almost too much direct instruction, please include more time for discussions […]”

^*^test segments that were categorized

Overview of the categories and subcategories of the open-ended questions. Statements (*n* = 57, k = 123) provided further information on the acceptance of ICCD and improvement suggestions for future semesters

### Evaluation of Tutors

Mean cognitive congruence and educational environment for all sessions were rated as excellent at 1.26 (*n* = 121) and 1.35 (*n* = 107) respectively. Most tutees ascribed the roles “information provider” (*n* = 106, 84.4%) and “facilitator” (*n* = 87, 69.6%) to their tutors. Several tutees also rated their tutors as “role models” (*n* = 68,54.4%) and “assessors” (*n* = 51, 40.8%).

### Learning outcome

CSA of learning outcomes revealed an increase in the ability to apply the correct diagnostic algorithm to a given case by 74.65% (*n* = 115). Ability to interpret the findings of diagnostic procedures increased by 70.31% (*n* = 114).

The end-of-course examination on internal medicine in the fall semester of 2020/21 consisted of 70 questions with a mean score of 85%. 30 (43%) questions have been previously discussed only during ICCD sessions, and 40 questions (57%) only during lectures. ICCD questions were answered with a higher score compared to lecture questions (90.3% vs. 82.4%, *p* = 0.074). Although not statistically significant, students’ overall performance measured as a grade in the end-of-course examination was improved by material produced during ICCD sessions.

#### Tutors

15 of 18 eligible tutors (83.33%) completed the survey. One tutor (author JR) conceived the questionnaire and was thus excluded to prevent potential bias.

Tutors rated the introductory seminar as helpful (mean 1.20) and indicated that the concept of the ICCD had been clearly communicated to them (mean 1.07). They did not report extreme difficulties conceiving a clinical case (mean 1.4) and indicated enjoying the process (mean 1.33).

## Discussion

This study aimed to report on an undergraduate students´ initiative to facilitate the core curriculum on internal medicine by developing and implementing the novel *Integrated Clinical Case Discussions* to train cognitive clinical skills relevant to the pertaining lecture. This information can help develop further student-led initiatives to address emergency remote teaching or other perceived curricular deficits with the expressed goal of training cognitive clinical skills.

The direct comparison of ICCDs and lectures versus emergency remote lectures alone revealed tutees´ subjective increased proficiency in clinical reasoning (determining diagnostic algorithm and interpreting findings). Similarly, students´ satisfaction levels rose. Tutees expressed positive feelings about being tutored by peers and high cognitive congruence.

We recorded increased participation rates in the second semester of ICCDs. The participation rate was 44.5% in the first and 71.0% in the second semester respectively. These participation rates merit special consideration, as the compulsory curriculum at TUM fulfils the legally required minimum number of classes and is supplemented with a broad range of voluntary courses (There are another 72 elective and extracurricular courses). This results in a competitive curricular environment in which students may be less intent on yet another learning opportunity, though the ICCDs are the only course covering the full spectrum of the lectures on internal medicine. The above-mentioned and increasing participation rates indicate that there is a target group that welcomes the offer of ICCDs, especially in the second semester on the cardiovascular and hematologic systems. We conclude, that a peer-moderated ICCD in response to emergency remote teaching can improve students´ subjective satisfaction level with learning opportunities and is in line with previous research [36]. Student satisfaction is important to consider, as it is one of the five pillars of Quality Online Education [37] and is positively correlated with student performance [38].

Our results support other studies highlighting the effectiveness of peer-teaching [39, 40] and CCD [41, 42] in teaching cognitive clinical skills. We found that students attending ICCDs in addition to the lecture benefitted from a gain in skill, overall satisfaction, motivation, and knowledge. This aligns with the ICCD´s goal of generating added value to the core curriculum.

Second, we conclude empowering students to organize and execute courses provides an effective way to create custom-tailored and widely accepted teaching formats. The excellent ratings of subjective learning outcomes, educational environment, and cognitive congruence support the notion that student leadership can be useful for curricular development [36, 43, 44].

We described the human and time resources for preparing one ICCD session. With student teachers contributing the most hours to an ICCD we are aware that additional teaching responsibilities might act as an additional stressor on tutors. However, our results suggest that tutors enjoy the process, feel well instructed and mentored in the workflow we proposed. Similarly, previous research has highlighted the benefits of being a peer teacher [10, 45]. Furthermore, students who agree to tutor have been shown to have the necessary resources to cope with the additional stress at their command [46].

It has been repeatedly demonstrated that voluntary courses receive better feedback than compulsory courses [47]. This study was limited by the ICCD´s voluntary nature, too. Selection bias in the evaluation may be introduced by the self-selection of students who are highly motivated to attend an ICCD session on top of the lecture in comparison to those who attended the general lecture alone. The modest overall response rate of 32% also suggests that certain opinions are likely to be overrepresented while others may be missing. However, with the respondent demographics reflecting the general student population at TUM, we believe our study provides worthwhile data. Response rates of approximately 30% have been reported before in the context of voluntary peer teachings [21]. A study by Bahous et al. (2018) suggests that the reliability between voluntary questionnaires with a low response rate and compulsory questionnaires with a high response rate is comparable [48]. To allow for a more comprehensive interpretation of results we also reported the maximum number of possible and de-facto attendances as demonstrated earlier [33]. The study design does not allow for a follow-up to assess the long-term impact on knowledge, skill, and attitude. Since our findings are based on data from one medical school in Germany they cannot be extrapolated to other medical schools without further consideration. However, the German model of medical education being common in Europe, we have reason to believe that study populations at other medical schools may be similar and our findings of value to their curricular designers [49].

## Conclusion

Empowering students to design their own add-on learning opportunities can improve learning outcomes, teach clinical reasoning skills beyond the scope of the core curriculum and increase satisfaction ratings with learning opportunities. We believe that our concept provides an easy-to-implement and up-scalable format to alleviate pressure on faculty staff and physicians with teaching capabilities for other schools, too.

For future optimization, we propose to advance the beneficial effect of social and cognitive congruence by inviting lecturers to facilitate ICCD sessions in person as we are now planning at TUM for the fall semester of 2022/23. This ultimately leads to a triangularized teaching format in which a student-tutor moderates the discussion, lecturers support discussions with more in depth-knowledge and clinical experience, and tutees engage in an instructive discussion.

## Data Availability

The datasets during and/or analyzed during the current study are available from the corresponding author upon reasonable request.

## Appendix

Appendix Table 1. Cognitive Congruence and Educational Environment. The table gives an overview of the items used to compute the cognitive congruence between tutors and tutees using the mean of section “1. Cognitive Congruence”, where 1 denotes the greatest and 5 the lowest level of tutees´ satisfaction. Tutees´ perception of the educational environment was computed by calculating the mean of the items in section “2. Educational Environment”. Cronbach´s alpha for cognitive congruence was 0.76; for educational environment 0.83.

**Table:**

	Mean	Mode	Std. Deviation	participants
1. Cognitive Congruence				
The tutor states learning objectives clearly	1.25	1.00	0.49	292
The tutor stresses relevant points	1.19	1.00	0.43	293
The tutor was able to explain complex matters clearly	1.23	1.00	0.46	293
The tutor acknowledges challenges	1.31	1.00	0.54	291
The tutor answers questions clearly	1.37	1.00	0.68	293
2. Educational Environment				
The tutor incentivizes participation	1.37	1.00	0.63	290
The tutorial has helped me connect new input to prior knowledge	1.23	1.00	0.46	291
The tutor creates space for practicing the contents of the tutorial	1.27	1.00	0.49	292
The atmosphere was pleasant and supportive	1.16	1.00	0.39	293
I feel it is okay to make a mistake	1.47	1.00	0.72	293
The tutor and other tutees showed respect and appreciation for my contributions	1.41	1.00	0.76	277
If at all, I was criticized respectfully	1.32	1.00	0.61	280

## Abbreviations

SARS-CoV2: Severe Acute Respiratory Syndrome Coronavirus Type 2
TUM: Technical University of Munich
PAL: Peer-assisted learning
ICCD: Integrated Clinical Case Discussion
GMTF: German Medical Training Framework
CCD: Clinical Case Discussion
SEEQ: Student´s Evaluations of Educational Quality Questionnaire
CSA: Comparative Self Assessment
